# Validation and Normative Data of the Spanish Version of the Rey Auditory Verbal Learning Test and Associated Long-Term Forgetting Measures in Middle-Aged Adults

**DOI:** 10.3389/fnagi.2022.809019

**Published:** 2022-02-09

**Authors:** Vanessa Alviarez-Schulze, Gabriele Cattaneo, Catherine Pachón-García, Javier Solana-Sánchez, Josep M. Tormos, Alvaro Pascual-Leone, David Bartrés-Faz

**Affiliations:** ^1^Institut Guttmann, Institut Universitari de Neurorehabilitació adscrit a la UAB, Badalona, Spain; ^2^Departament de Medicina, Facultat de Medicina, Universitat Autònoma de Barcelona, Bellaterra, Spain; ^3^Fundació Institut d’Investigació en Ciències de la Salut Germans Trias i Pujol, Badalona, Spain; ^4^Departamento de Ciencias del Comportamiento, Escuela de Psicología, Universidad Metropolitana, Caracas, Venezuela; ^5^Universitat Autònoma de Barcelona, Bellaterra, Spain; ^6^Hinda and Arthur Marcus Institute for Aging Research and Deanna and Sidney Wolk Center for Memory Health, Hebrew SeniorLife, Boston, MA, United States; ^7^Department of Neurology and Harvard Medical School, Boston, MA, United States; ^8^Departament de Medicina, Facultat de Medicina i Ciències de la Salut, Institut de Neurociències, Universitat de Barcelona, Barcelona, Spain

**Keywords:** episodic memory, long-term forgetting, Memory and Learning test, validation study, neuropsychological test, RAVLT validation

## Abstract

Rey Auditory Verbal Learning Test (RAVLT) is an episodic memory helpful measure to detect changes associated with abnormal aging. There is a lack of RAVLT validation and normalization studies in Spain. The aim was to determine its psychometric properties and explore long-term forgetting (LTF) performance through 1-week delayed recall under three different modes of administration. The RAVLT was administered to 602 cognitively healthy volunteers, aged between 41 and 65 years, of whom 251 completed the LTF assessment. Findings reveal a factorial structure of four components, with satisfactory goodness of fit, and adequate convergent and divergent validity. We also demonstrated the differential effect of three methodologies used in LTF assessment, supporting that test expectancy positively influences long-term storage. Finally, normative data were generated according to age, sex, and education. The test, including the LTF measure, is a promising tool to estimate memory in middle-aged adults and develop predictive brain aging models.

## Introduction

Episodic memory (EM) defined by Tulving (2002) as a neurocognitive system, particular and different from other memory domains, that enables to store specific experiences in terms of what happened and where and when the events happened (Pause et al., 2013) is considered a highly sensitive indicator of incipient brain pathology. Its decline has been considered as a predictor of cognitive impairment toward dementia, even 10 years prior to the clinical diagnosis (Estévez-González et al., 2003; Pause et al., 2013; Boraxbekk et al., 2015; Lu et al., 2018).

Given its complexity, heterogeneity in EM performance has been found in various clinical populations, and the definition and quantification of EM decline in aging remain a questioned topic (Pause et al., 2013; Nyberg and Pudas, 2019). For this reason, the existence of adequately validated and standardized psychometric tests for memory measurement in different modalities (verbal and visual) is essential to identify healthy people, higher performers, or maintainers (successful memory aging), as well as those subjects with memory decline who show a negative rate of change in longitudinal measurements (Nyberg and Pudas, 2019).

Within the mnesic function, LTF is a dimension that has been less studied (Baddeley et al., 2019). Accelerated long-term forgetting (ALF) is defined as a phenomenon by which memories that are encoded and maintained during intervals of approximately 30 min are then forgotten faster than expected over delays of days/weeks (Elliott et al., 2014). However, this definition is currently under review because there are no unique methods for calculating the ALF score (Butler et al., 2019).

The study of ALF shows high potential to improve Alzheimer’s Disease (AD) prediction, and it has been strongly recommended to include it within longitudinal research (Tort-Merino et al., 2021a). Recent findings have suggested that LTF measures are much more sensitive to assess earlier pathological memory decline associated with mild cognitive impairment (MCI) and AD (Geurts et al., 2015). In fact, Wearn et al. (2020) found that the long-term delay period can improve the detection of subjects likely to decline during the following year. Also, there is evidence of the relation between ALF and early markers of AD, including subjective complaints, autosomal dominant AD mutation carriers, APOE E4 carriers, and abnormal levels of CSF Aβ42 and CSF Aβ42/ptau ratio (Tort-Merino et al., 2017, 2021a,2021b; Reiman, 2018; Weston et al., 2018; Zimmermann and Butler, 2018; Butler et al., 2019).

There is an urgent need for reliable, valid, and standardized LTF measures (Elliott et al., 2014; Baddeley et al., 2019; Mayes et al., 2019), considering that most of the long-term episodic memory tests usually limit retrieval to 20/30 min.

The RAVLT is a widely used episodic verbal memory test that measures encoding, consolidation, storing, and retrieval of verbally learned content (Schmidt, 1996; Schoenberg et al., 2006). Previous research suggests that the RAVLT is helpful to predict the progress of MCI to dementia (Estévez-González et al., 2003; Schoenberg et al., 2006; Drolet et al., 2014; Marchand et al., 2017; Moradi et al., 2017).

The RAVLT is a 15-unrelated-word verbal list-learning task (Rey, 1964; Schmidt, 1996). Different versions of this instrument have been developed, with variations in its application procedures (Estévez-González et al., 2003; Lezak et al., 2012; Bezdicek et al., 2014; Cavaco et al., 2015) that need to be considered for the comparison between studies, particularly for normative data selection (Messinis et al., 2007).

The version that we aim to validate is frequently used in Spain for clinical practice and research (Perea Bartolomé et al., 2000; Estévez-González et al., 2003; García-Rudolph et al., 2020; Albu et al., 2021; Vaqué-Alcázar et al., 2021) and consists of five initial learning trials (Trials I–V) that imply attention, encoding, and reflect the ability to learn context-free auditory verbal stimuli over repeated practice. After a period of 20-min, Delayed Recall (Trial VI) is requested, followed by a recognition task (Trial VII), allowing to assess evocation and storage processes (Schoenberg et al., 2006; Lezak et al., 2012; Cavaco et al., 2015; Puerta Lopera et al., 2018). On the other hand, other versions also contain an interference list of 15 words (list B) after trials 1–5, which measures the interference effect (Schmidt, 1996; Lezak et al., 2012).

The classic RAVLT quantification, the total number of words recalled in each trial, has been shown to reflect specific patterns in healthy and clinical populations (Libon et al., 2015; Lu et al., 2018). However, some authors highlight the relevance of taking executions errors (perseverations and intrusions) into account when analyzing memory tasks because they are useful to characterize abnormal brain aging (Bezdicek et al., 2014; Ferreira Correia and Campagna Osorio, 2014; Libon et al., 2015; Montero and Cáceres, 2017; Weitzner et al., 2020).

The RAVLT has been previously standardized and validated in different languages, with different health and clinic populations (Schoenberg et al., 2006; Messinis et al., 2007; Fichman et al., 2010; Vakil et al., 2010; Bezdicek et al., 2014; Speer et al., 2014; Cavaco et al., 2015; Lavoie et al., 2018). Although there are studies with Hispanic populations (Marqués et al., 2013; Ferreira Correia and Campagna Osorio, 2014; Sánchez-Nieto et al., 2016; Puerta Lopera et al., 2018), there have been no published validation and standardization studies in recent decades with a representative healthy middle-aged Spanish sample, and its adapted and validated versions do not formally include LTF measurement.

Moreover, even if LTF of this test has been previously used in experimental studies, the procedures employed were not homogeneous (Mameniskiene et al., 2006; Butler et al., 2007; Atherton et al., 2019; Savage et al., 2019). In this vein, Elliott et al. (2014) introduced some methodological issues regarding LTF assessment. The main problem is the need to eliminate or minimize the possibility of rehearsal during delays. To solve this, Elliott et al. (2014) mentioned that some researchers have decided not to forewarn participants about a deferred evocation requirement (Weston et al., 2018; Wearn et al., 2020; Tort-Merino et al., 2021a). However, this method could not be the most appropriate within the clinical practice or longitudinal studies. If later assessments are needed, the comparisons between longitudinal points in time would be biased due to the effect of the test expectation on the consolidation process. Test expectation refers to the assumption of the “future relevance” of learned information given the warning of a long-term delayed recall (Shimizu, 1996; Wamsley et al., 2016).

Alternatively, other authors assumed a procedure that consists of asking the participants intentionally not to rehearse but explicitly did not forewarn them about the delayed retrieval after days/weeks; they were only informed that they would receive a call to complement the evaluation (Butler et al., 2007; Muhlert et al., 2010; Hoefeijzers et al., 2013; Savage et al., 2019). Nevertheless, as in the procedure described above, subjects may predict that they will undergo an LTF testing again in longitudinal studies or clinical follow-up assessments.

In some other cases, the procedure described by the researchers is not clear (Davidson et al., 2007; Atherton et al., 2019). Considering that in follow-up measures the subjects could predict that they will be asked for a delayed recall, another possible application modality would be to inform them about the LTF probe requiring them not to practice. However, we hypothesize that test expectations and knowing that learned content will be asked could affect LTF performance.

It is essential to discuss these methodological concerns because there is evidence that rehearsal may decrease LTF (Elliott et al., 2014). Still, the potential effect of the different application modalities described above is unknown. Thus, possibly each application version would require specific normative data.

In conclusion, given the importance of assessing episodic memory as a preclinical indicator of abnormal aging, our main objectives were: (1) to determine the RAVLT validity and develop population-specific normative data in a Spanish sample of healthy individuals aged between 41 and 65 years; (2) to explore sample performance on the proposed LTF measure with a 1-week delayed recall using RAVLT under three different administration modalities.

## Materials and Methods

### Participants

This study was conducted using data collected in 2019 on a subset of the participants enrolled in the in-person assessment of the Barcelona Brain Health Initiative (BBHI), a longitudinal prospective population-based cohort study (Cattaneo et al., 2018).

#### Validation and Normative Sample

The validation sample consisted of 602 volunteers (280 women, mean age = 53.50, *SD* = 6.96, age range, 41–65, mean years of education = 17.21, *SD* = 3.74, 95% Caucasians) who had completed the entire neurocognitive assessment protocol at the moment of the analysis. Most of the participants were Catalonia residents (96.01%) and only 3.99% came from other areas within Spain. About 95.01% of our participants were Catalan-Spanish bilinguals (4.99% were only Spanish speakers).

#### Long-Term Forgetting Sample

Among the total participants who completed the in-person neuropsychological assessment, 251 subjects also completed the LTF assessment with a 1-week delayed recall. The rest of the sample could not be contacted by telephone in the scheduled period. These participants were pseudo-randomly assigned to one of three groups to assess and compare three different administration procedures. Groups were balanced for sex, age, and education.

Following BBHI exclusion criteria, the participants with a history or current diagnosis of neurological or psychiatric disease (*n* = 13), TBI with loss of consciousness (*n* = 4), substance abuse/dependence (*n* = 9), or treatment with psychopharmacological drugs (*n* = 9) were excluded from the study. Also, we did not include the participants with objective deficits in neuropsychological tests (*n* = 15) (see the Section “Procedures and Materials”) and those whose assessment was significantly interfered with (*n* = 4) for a variety of reasons (external interruptions, prior familiarity with the test, and task abandonment). The participants provided explicit informed consent, and the protocol was approved by the Comitéd’ Èticai Investigació Clínica de la Unió Catalanad’ Hospitals (Cattaneo et al., 2018).

### Procedures and Materials

The RAVLT was administered according to the standardized procedure during the BBHI cognitive assessment session (Cattaneo et al., 2018). Its administration lasted 30–35 min, including the lapse required to measure delayed recall. It is crucial to highlight that no other memory tests were applied between the RAVLT trials. The version used consists of the oral presentation of a list of 15 unrelated Spanish words (Perea Bartolomé et al., 2000; Estévez-González et al., 2003). The application procedure started with the initial learning (the encoding phase) of a 15-word list, which is read by the investigator at the rate of one word per second, followed by an immediate recall. The examinees should repeat all the words they could remember, regardless of the order. This operation was continuously repeated five times (I–V trial). After 20–25 min, the participants were then requested to retrieve as many list words (delayed recall). Finally, a recognition task (recognition) required the examinees to identify the words practiced within a broader list that includes new content.

During the BBHI cognitive assessment session (Cattaneo et al., 2018), a comprehensive neuropsychological tests battery was administered in the following fixed order: S-FNAME Exam (Alegret et al., 2015; Alviarez-Schulze et al., 2022) direct and inverse digit spans (Peña-Casanova et al., 2012), Trail Making Test parts A and B (TMT-A and TMT-B, respectively) (Peña-Casanova et al., 2012), phonemic and semantic fluency tasks (Peña-Casanova et al., 2012), Matrix Reasoning subtest from Wechsler Adult Intelligence Scale-Fourth Edition (WAIS-IV) (Wechsler, 2012), Rey Auditory-Verbal Learning Test (RAVLT; Schmidt, 1996), Block Design subtest from WAIS-IV (Wechsler, 2012), Letter-Number Sequencing (Peña-Casanova et al., 2012), Digit-Symbol Substitution Test and Cancelation subtests from WAIS-IV, and Corsi block-tapping test (Peña-Casanova et al., 2012). The cognitive assessment session was conducted by two expert neuropsychologists and lasted approximately 90 min.

At the end of the in-person testing, the participants received one of the three different instructions about the 1-week call, as we mentioned above, to measure LTF through the RAVLT word list free recall and recognition task. Specifically, the LTF procedure modalities were:

• The participants of Group I (*n* = 83) were warned that they would receive a phone call to collect some general information, without any reference that what will be requested is related to neuropsychological evaluation.
• The participants of Group II (*n* = 85) were warned about a call to answer some questions related to the neuropsychological assessment conducted during the in-person session. Still, they were not specifically informed about the LTF measure, and, however, they were explicitly asked not to rehearse material or any of the activities included in the neuropsychological assessment.
• The participants of Group III (*n* = 83) were explicitly forewarned about the 1-week delayed retrieval of the RAVLT word list, and they were directly and precisely requested not to practice the word list.

Previous LTF research (Weston et al., 2018; Zimmermann and Butler, 2018) has fixed a criterion of the accuracy of 80% of acquired information during initial learning. However, in this study, we followed one of the standardized versions of the RAVLT (Perea Bartolomé et al., 2000; Estévez-González et al., 2003) that consists of five initial learning trials, considering that we aimed to validate the test and generate its normative tables for the Spanish population.

To determine if the participants did rehearse after the testing session, we asked during the phone call if they wrote down the information, the words were spontaneously evoked, or voluntarily practiced during the week. We excluded those who answered affirmatively. Only one subject of Group II reported having practiced, and four participants of Group III were excluded for this reason.

### Data Analysis

Statistical analyses were executed using SPSS version 22.0 (Statistical Package for Social Sciences, Chicago, IL, United States). Statistical significance was determined when *p* < 0.05 for all the inferential analyses.

#### Validation Data Analysis

The distribution of sociodemographic variables (age ranges, educational level, and sex) of the validation sample was estimated. Years of education were measured by explicitly asking the volunteers to inform the total time of formal education, counting from the beginning of obligatory education in Spain (primary school). Spanish educational system includes elementary/primary school (6 years), obligatory secondary school (4 years), and baccalaureate/high school or middle-grade vocational training (2 years). Higher education comprises undergraduate degrees (4 years) and post-graduate degrees (specialization, master’s, and Ph.D. programs).

Descriptive analyses were performed for RAVLT trials and errors (repeated words, intrusions, and repeated intrusions). We included complementary scores: total recall (sum of trials I, II, III, IV, and V) and the learning over trial (LOT) score corresponding to total learning corrected for an immediate word span [Total Recall–(5 × Trial I)]. The latter estimates better the improvement across trials than other scores (Vakil et al., 2010; Marqués et al., 2013; Cavaco et al., 2015). Also, we calculated the forgetting rate [1 - (delayed recall/Trial V) and multiplied by 100].

We ran an exploratory factor analysis (EFA) using principal component analysis and OBLIMIN rotation. Confirmatory factor analysis (CFA) was carried out using IBM SPSS AMOS to verify the fit of the factor structures obtained from the EFA to choose the most plausible model avoiding indeterminacy bias, following the rival model strategy proposed by Hair et al. (1999). To examine, the goodness of fit considered the absolute, incremental, and parsimonious fit indices: Chi-square (χ^2^), Normed Chi-square (χ^2^/df), Goodness of Fit Index (GFI), Adjusted Goodness Fit Index (AGFI), Root Mean Square Error of Approximation (RMSEA), Normed Fit Index (NFI), Tucker-Lewis Index (TLI), parsimonious normed fit index (PNFI), and Parsimony Goodness-of-Fit Index (PGFI) (Hair et al., 1999).

Convergent validity was calculated using Pearson correlation coefficients between RAVLT scores (Immediate Total Recall and Delayed Recall) and S-FNAME scores (subtotal scores of face-name and face-occupation association: S-FNAME FN-N and S-FNAME FN-O). Divergent validity was assessed using Pearson correlation coefficients with non-memory tests: TMT-A, TMT-B, Matrix Reasoning, and Block Design subtests.

#### Normative Data Analysis

The correlation between demographic variables and RAVLT scores was calculated using Pearson correlation coefficients. We carried out multiple linear regression analyses to examine the contribution of sex, age, and education on RAVLT performance.

Analysis of variance (ANOVA) was used to determine the effect of sociodemographic variables, taking into account those regression models that explained at least 5% of the total variance of RAVLT scores (Peña-Casanova et al., 2012; Ferreira Correia and Campagna Osorio, 2014). Therefore, ANOVA was calculated to determine the effect of sex, age ranges (41–48, 49–57, ≥58 years), and education level (<16 years of education ≥ 16 years of education) on RAVLT measures.

The configuration of the age groups was data driven and corresponds to the terciles of the sample to guarantee a similar sample size within the bands. Specifically, it was obtained after multiple comparisons between different ranges to guarantee that the resulting groups reflected significant differences in RAVLT scores, instead of arbitrarily dividing age (Ferreira Correia and Campagna Osorio, 2014; Alviarez-Schulze et al., 2022). The division of the groups by years of education corresponds to the level of higher education (16 years), considering the distribution of our sample.

Finally, the RAVLT norms were developed following the traditional norming method, considering the combination of those demographic variables that demonstrated a significant effect. Additionally, we estimated demographically adjusted based-regression norms that provide the resulting *z*-scores metric obtained through the statistical procedure described below (Bezdicek et al., 2014; Cavaco et al., 2015; Kormas et al., 2018; Lavoie et al., 2018):

1. From the regression equation obtained by multiple regression analyses, we calculated the predicted raw scores adjusted for sociodemographic variables (age, sex, and years of education) that resulted statistically significant. A case wise diagnosis identified possible outliers, and we confirmed the regression assumptions were met.
2. The residuals were calculated by subtracting the predicted value from the observed raw score.
3. Finally, we standardized the residuals by dividing them by the standard error of the estimate (SEE) of the regression line. The *z*-scores obtained are interpreted using a *Z* distribution table to determine the examinees’ performance compared to their normative group.

#### Long-Term Forgetting Data Analysis

The distribution of sociodemographic characteristics of the LTF sample according to sex, age ranges (41–49, 50–57, 58–65 years), and educational level (<16 and ≥16 years) was calculated for each group under the different administration modalities proposed.

Descriptive analyses for each group were performed for 1-week delayed measures: 1-week Delayed Recall, 1-week Delayed Recognition, 1-week intrusions, and 1-week Forgetting Rate. One-week Forgetting Rate refers to the ratio of information loss between the 25 min Delayed Recall and 1-week Delayed Recall scores. It was calculated using the formula: [1 - (1-week Delayed Recall/25-min Delayed Recall)] × 100.

We ran a one-way ANOVA to compare group performance on the cognitive tests administered during the neuropsychological assessment session, including the RAVLT measures. These analyses were performed to ensure homogeneity between groups.

A mixed ANOVA was conducted to determine whether changes in the number of words evoked are a result of the interaction between the administration modality and the time lapse of delayed recall. This analysis will determine changes between the 25-min and 1-week delayed recall measures that depend on the application modality corresponding to the groups (Interaction Effect).

Finally, we ran linear regression analyses for each group to explore the possible influence of sociodemographic variables on the LTF measures (1-week Delayed Recall, 1-week Recognition, and 1-week Forgetting Rate).

## Results

### Rey Auditory Verbal Learning Test Validation

The distribution of sociodemographic variables (age ranges, education, and sex) of the validation sample is presented in Table 1. Distribution by sex was homogeneous according to a one-sample binomial test (*p* = 0.095). Also, the number of males and females within each age ranges [χ*^2^* (2) = 0.165; *p* = 0.921] was uniform.

**TABLE 1:** Frequencies of demographic characteristics of a validation sample.

	Age range	41–49 years		50–57 years		58–65 years		Total	
		*n* = *204*		*n* = *195*		*n* = *203*			
		*n*	%	*n*	%	*n*	%	*n*	%
Years of Education	0–15	48	23.5	53	27.2	80	39.4	181	30.1
	≥16	156	76.5	142	72.8	123	60.6	421	69.9
Gender	Women	94	46.1	93	47.7	93	45.8	280	46.5
	Men	110	53.9	102	52.3	110	54.2	322	53.5

An unequal sample distribution (*p* < 0.001) by educational level (<16 years of education ≥ 16 years of education) was observed. Also, the distribution of years of education was unbalanced between age bands, with a larger proportion of education below 16 years in the older individuals. However, the educational level was similar between males and females [χ*^2^* (1) = 0.74; *p* = 0.39].

Descriptive analysis for RAVLT trials, errors, and complementary scores (Immediate Total Recall, LOT, and Forgetting Rate) was carried out (see Table 2).

**TABLE 2:** Description of RAVLT, errors, and complementary scores.

	Min	Max	Mean	*SD*
I	2	13	6.26	1.77
II	5	15	9.62	2.19
III	5	15	11.27	2.09
IV	6	15	12.21	1.90
V	6	15	12.73	1.87
Immediate Total Recall	29	71	52.09	8.19
Delayed Recall	3	15	11.36	2.56
Recognition	8	15	14.41	1.06
Repeated words	0	18	4.85	3.59
Intrusions words	0	6	0.67	0.94
Repeated intrusions	0	8	0.33	0.92
LOT	3	40	20.81	6.45
Forgetting Rate	−25	62.5	11.09	13.96

*I, Trial I of Rey Auditory Verbal Learning Test (RAVLT); II, Trial II of RAVLT; III, Trial III of RAVLT; IV, Trial IV of RAVLT; V, Trial V of RAVLT. LOT, Learning Over Trial or Immediate Total Recall – (5 × Trial I).*

#### Construct Validity

Exploratory factor analysis (EFA) was carried out using principal component analysis and Oblimin rotation (Hair et al., 1999). The Kaiser–Meyer–Olkin measure of sampling adequacy (KMO = 0.868) and Bartlett’s test of sphericity (χ^2^ = 2922.23; *gl* = 45; *p* < 0.001) were satisfactory, and determinant of correlation matrix 0.007 tended to 0 as expected (Hair et al., 1999).

The EFA yielded three factors with an eigenvalue greater than 1.00. This solution explained 70.15% of the total variance. The 3-factor model showed that Factor 1 loads Trials I, II, III, IV, and V, Delayed Recall and Recognition; Factor 2 was related to Intrusions and Repeated intrusions words; and Factor 3 only loads repeated words, as presented in Table 3.

**TABLE 3:** Rey Auditory Verbal Learning Test factor structure obtained from EFA.

Rotated components matrix			
	Component		
	1	2	3
IV	0.87	–0.19	–0.03
III	0.87	–0.21	–0.01
Delayed Recall	0.85	–0.24	–0.13
V	0.84	–0.17	–0.08
II	0.84	–0.12	0.18
I	0.65	–0.07	0.24
Recognition	0.62	–0.18	–0.09
Repeated Intrusions	–0.14	0.88	0.02
Intrusions words	–0.24	0.87	0.09
Repeated words	0.00	0.05	0.95

*I, Trial I of Rey Auditory Verbal Learning Test (RAVLT); II, Trial II of RAVLT; III, Trial III of RAVLT; IV, Trial IV of RAVLT; V, Trial V of RAVLT.*

Considering the criteria of the Scree plot and the explained variance above 60%, a 2-factor model was found. One factor is related to RAVLT Trials, and the other includes errors (only Intrusions, repeated words did not load on any factor).

Finally, before carrying out CFA, we established an *a priori* factorial structure from a theoretical basis of the construct and previous findings (Vakil and Blachstein, 1993; Baños et al., 2005) that suggest one component associated with the Attention and Memory span (Trials I and II) and others related to Memory and Learning (Trials III, IV, V, Delayed Recall, and Recognition); we included a third dimension composed of Intrusions and a fourth component related to repeated words. Therefore, a four-factor model resulted.

Confirmatory factor analysis results reflected comparisons between absolute fit indicators, incremental fit measures, and parsimony of each rival model (see Supplementary Table 1 for details). All chi-squares reflected high values, contrary to expectations, but this indicator is not sensitive in the case of large samples. χ^2^/gl index seems less sensitive to the sample size; smaller magnitudes are considered a better fit. The 4-factor model showed lower χ^2^/gl, below 5, a cut-off point indicating an acceptable level (Hair et al., 1999).

All GFI indices were adequate (>0.9), although the 4-factor model reflected superior fit (GFI = 0.95). Concerning RMSEA, one of the most critical indicators, the 4-factor model was the only one that fell within the acceptable range below 0.08 (Hair et al., 1999; Batista and Coenders, 2000).

Regarding the incremental fit indicators, the 4-factor model showed an adequate value, near to 1; the other models obtained unsatisfactory values below 0.90. NFI and NNFI for all models reflected deficient values. Finally, parsimonious fit indices (PGFI and PNFI) of all models reflected unsatisfactory values. Therefore, these indices are not useful as a criterion to compare and complement the choice of the best fit model.

The most important and relevant index to select the best model is the absolute fit indices, especially RMSEA. Therefore, the 4-factor model, which is consistent with previous findings, is chosen to explain the factor structure of the RAVLT.

#### Convergent and Divergent Validity

To examine the convergent validity of the RAVLT, Pearson correlation coefficients (*r*) were calculated between RAVLT scores and the S-FNAME performance. Statistically significant (*p* < 0.01) associations were found with medium effect size. In addition, regarding divergent validity, we obtained Pearson correlation coefficients between RAVLT scores and non-memory measures. We found positive associations (*p* < 0.01) between RAVLT scores and TMT-A, TMT-B, Matrix Reasoning and Block Design (WAIS IV), but with small effect size and negative correlation (*p* < 0.01) with TMT-A and TMT-B (time measurements), with a small effect size as well (see Table 4).

**TABLE 4:** Correlation coefficients between RAVLT scores, memory and non-memory tests scores.

	S-FNAME	TMT-A	TMT-B	Reasoning Matrix	Block Design
Immediate Total Recall	0.43**	−0.21**	−0.22**	0.21**	0.17**
Delayed Recall	0.37**	−0.19**	−0.18**	0.20**	0.14**

** p < 0.01.

*S-FNAME, Spanish FNAME Exam total score; TMT-A, Trail Making Test Part A; TMT-B, Trail Making Test Part B.*

To confirm evidence of divergent validity, we used Steiger’s *z* statistic to compare correlations between RAVLT scores and non-memory tests scores with those obtained by measuring convergent validity. Correlation between the RAVLT performance (Immediate Total Recall and Delayed Recall) and TMT scores was lower than the correlation between the RAVLT and both S-FNAME scores (Steiger’s *z* = −4.46, *p* < 0.001, Steiger’s *z* = −3.57, *p* < 0.001; Steiger’s *z* = −4.37, *p* < 0.001; Steiger’s *z* = −3.85, *p* < 0.001). Likewise, Matrix Reasoning and Block Design reflected lower correlation coefficients with the RAVLT performance than the coefficients between the RAVLT and both S-FNAME scores (Steiger’s *z* = −4.29, *p* < 0.001; Steiger’s *z* = −3.39, *p* < 0.001; Steiger’s *z* = −5.29, *p* < 0.001; Steiger’s *z* = −4.58 *p* < 0.001).

### Normative Data

To explore possible associations between demographic variables and the RAVLT measures, we calculated Pearson correlation coefficients. Negative correlations between age and RAVLT scores and positive association between years of education and test performance were found (*p* < 0.01) (see Supplementary Table 2 for details).

Thus, we ran multiple regression analyses to confirm the contribution of sex, age, and years of education as predictors of RAVLT scores (see Table 5). Findings revealed a significant contribution of these variables to the variance of RAVLT Immediate Total Recall (*p* < 0.001) and Delayed Recall (*p* < 0.001). Nonetheless, the regression model for Recognition measure only explained 4.1% and for Forgetting Rate only explained 2%. Additionally, regarding error measures, a very low contribution to the total variance (2%) of Repeated words (the model explained *p* < 0.01) and regression models for Intrusions (*p* = 0.14) and Repeated Intrusions (*p* = 0.07) were not significant.

**TABLE 5:** Contribution of age, gender, and education on RAVLT scores.

Measure	Predictor	Standardized β	*T*	*p* value	*R* ^2^
Immediate Total Recall	Age	–0.24	–6.31	<0.001	0.13
	Gender	–0.22	–5.65	<0.001	
	Education (years)	0.14	3.54	<0.001	
Delayed Recall	Age	–0.19	–4.84	<0.001	0.09
	Gender	–0.19	–3.70	<0.001	
	Education (years)	0.15	4.17	<0.001	
Recognition	Age	–0.10	–2.47	0.01	0.04
	Gender	–0.0.9	–2.15	0.03	
	Education (years)	0.15	3.66	<0.001	
Forgetting Rate	Age	0.10	–2.54	0.01	0.02
Repeated words	Age	0.05	1.21	0.23	
	Gender	–0.14	–3.33	<0.001	0.02
	Education (years)	0.02	0.51	0.61	

The effects of age (divided into ranges: 41–49, 50–57, and 58–65 years), educational level (<16 and ≥16 years of education), and sex were calculated using ANOVA with Bonferroni correction since the condition of equality of variances was demonstrated using Levene test.

Age [*F*(2,590) = 12.26, *p* < 0.001, η^2^ = 0.04], sex [*F*(1,590) = 21.10, *p* < 0.001, η^2^ = 0.04], and education [*F*(1,590) = 11.38, *p* < 0.001, η^2^ = 0.02] showed significant impact on Immediate Total Recall. *Post hoc* analyses revealed differences between all age groups (*p* < 0.001), reflecting that as the age range increases, the performance decreases significantly. Men, older, and less educated individuals obtained a lower Immediate Total Recall score.

Regarding Delayed Recall, sex [*F*(1,590) = 18.71, *p* < 0.001, η^2^ = 0.03], age [*F*(2,590) = 7.29, *p* < 0.001, η^2^ = 0.02], and education [*F*(1,590) = 8.82, *p* < 0.01, η^2^ = 0.02] groups differed significantly in terms of their performance, reflecting the same pattern described for Immediate Total Recall scores. *Post hoc* analyses showed statistically significant difference between the youngest (41–48 years old) and oldest (58–65 years old) groups (*p* = 0.003) and between the middle-aged range (49–57 years old) and the oldest group. Interaction effects between sociodemographic variables were not found for any of the RAVLT measures.

Then, we stratified and described Immediate Total Recall and Delayed Recall scores by sex, age, and educational level according to ANOVA results (see Supplementary Table 3) to generate traditional population-specific norms of healthy Spanish individuals aged between 41 and 65 (see Tables 6–9). Stratified percentile tables were not generated for Recognition, Forgetting Rate, and Repeated word measures since the regression models explained less than 5% of the total variance. Likewise, the regression models for other error scores were not statistically significant; therefore, normative tables were developed for the entire sample distribution (Table 10).

**TABLE 6:** Normative data for women performance on RAVLT Immediate Total Recall.

	Women					
	41–49 years		50–57 years		58–65 years	
*Percentile*	<16 yrs educ	≥16 yrs educ	<16 yrs educ	≥16 yrs educ	<16 yrs educ	≥16 yrs educ
2	37	44	35–36	33–37	29–32	40
5	38–41	45–46	37–41	38–43	33–39	41
10	42–44	47–49	42–43	44–45	40–41	42–43
15	45	–	44	46	42–43	44–45
20	46	50	45–46	47–48	44–47	–
25	47	51–52	47–48	49	–	46–47
30	48	53	49	50	48	48
35	49	54–55	50	51	–	49
40	–	56	51	52	49	50–51
45	50	57	52–53	–		52
50	51	58	54	53–54	50–53	53
55	52–53	59	–	55	54	54
60	54	–	55	56	55	55
65	55–56	60	56	57–58	–	56
70	57	–	–	59	56	–
75	58–59	62–63	57	60	–	57–58
80	–	64	58–59	61–62	57–58	59
85	60	65	60	63	59–61	60–62
90	61–62	66	61–65	64	62–64	63–67
95	63	67–68	66–67	65–67	65	68–69
98	–	≥ 69	–	≥68	–	≥ 70

*<16 years of education, less than 16 years of education; ≥16 years of education, 16 years of education or more.*

**TABLE 7:** Normative data for men performance on RAVLT Immediate Total Recall.

	Men					
	41–49 years		50–57 years		58–65 years	
*Percentile*	<16 yrs educ	≥16 yrs educ	<16 yrs educ	≥16 yrs educ	<16 yrs educ	≥16 yrs educ
2	39	35–38	–	33–36	33	33–35
5	40–41	39–42	37–39	37–39	34–36	36
10	42–44	43–46	40	40–41	37–38	37–38
15	45	47	41	42	39	39–40
20	46–48	48	42	43–44	40–41	41
25	–	49	–	45–46	–	42–43
30	49	50–51	43	47	42	44
35	50	52	44	48	–	45
40	–	–	45	49	43	46
45	51	53	46–47	50–51	–	47
50	52	54	48	–	44	48
55	53	55	49	52	45–46	49
60	54	–	50	53–55	47–48	50
65	55–56	56	51–52	–	49	51–52
70	57	57–58	53	56	50	53–54
75	–	59–61	54	57–59	51–53	55
80	58	62	55	60–61	54	56–57
85	59–60	63	56–59	62	55–56	58
90	61–64	64–65	60–64	63	57–61	59–60
95	65	66	65	64–65	62	61
98	–	≥67	–	≥66	–	≥62

*<16 years of education, less than 16 years of education; ≥16 years of education, 16 years of education or more.*

**TABLE 8:** Normative data for women performance on RAVLT Delayed Recall.

	Women					
	41–48 years		49–57 years		58–65 years	
*Percentile*	<16 yrs educ	≥16 yrs educ	<16 yrs educ	≥16 yrs educ	<16 yrs educ	≥16 yrs educ
2	5	6	–	6	–	6
5	6	7–8	8	8	5–6	7
10	7–8	9–10	9	9	7–8	8
20	9	11	10	10	9	9
30	10	12	–	11	10	10
40	–	–	11	–	11	11
50	11	13	–	12	–	–
60	–	–	12	13	12	–
70	12	14	13	–	13	12
80	13	–	14	14	14	13
90	14	–	–	–	–	14
95	15	15	15	15	15	15

*<16 years of education, less than 16 years of education; ≥16 years of education, 16 years of education or more.*

**TABLE 9:** Normative data for men performance on RAVLT Delayed Recall.

	Men					
	41–48 years		49–57 years		58–65 years	
*Percentile*	<16 yrs educ	≥16 yrs educ	<16 yrs educ	≥16 yrs educ	<16 yrs educ	≥16 yrs educ
2	6	5–6	5	5	–	4
5	7	7	6	6	6	5–6
10	8	8–9	7	7–8	7	7
20	9	10	8	9	–	8
30	–	11	–	10	8	9
40	10	–	–	–	9	–
50	11	12	9	11	–	10
60	12	13	10–11	12	10	11
70	–	–	12	13	11	12
80	13	14	13	14	12	13
90	14	–	14	–		14
95	15	15	15	15	≥ 13	15

*<16 years of education, less than 16 years of education; ≥16 years of education, 16 years of education or more.*

**TABLE 10:** Normative data for Recognition, Errors, Learning Over Trial (LOT), and Forgetting Rate on RAVLT.

Percentiles	Recognition	Repeated	Intrusions	Repeated Intrusions	LOT	Forgetting Rate
2	≤11	≥14	≥3	≥4	7–9	≥45
5	12	11–13	2	2–3	10–12	44–37
10	13	10	–	1	13–15	36–31
20	–	8–9	–	–	16	21–30
30	14	6–7	–	0	17–18	17–20
40	–	5	1		19–20	13–16
50	15	4	0		21	9–12
60		–			22–23	7–8
70		3			24–25	–
80		2			26–28	0
90		1			29–31	−1 to −7
95		0			32–34	−8 to −10
98					≥35	≤-11

*LOT, Learning Over Trial or Immediate Total Recall - (5 × Trial I).*

It is important to note that the 15th percentile corresponds to mild impairment (a score more than one SD below the mean), and the 2nd percentile is the cut-off point for a significantly impaired performance (two SDs below the mean).

Additionally, we estimated demographically adjusted equations to calculate RAVLT *z* scores through the regression-based norming method. We adjusted scores for age, sex, and years according to the regression models for Immediate Total Recall and Delayed Recall (see details in Supplementary Table 4).

### Long-Term Forgetting Assessment

Sex, age ranges (41–49, 50–57, and 58–65 years), and educational level (<16 and ≥16 years) are represented in Table 11. We found the distributions by sex [χ*^2^* (2) = 0.29; *p* = 0.98], age range [χ*^2^* (4) = 5.82; *p* = 0.21], and educational level [χ*^2^* (2) = 0.254; *p* = 0.88] were similar between the three groups. Thus, they are homogeneous according to their sociodemographic characteristics.

**TABLE 11:** Demographic characteristics of the long-term forgetting (LTF) sample.

		Administration modality					
		Group I *n* = *83*		Group II *n* = *85*		Group III *n* = *83*	
		*n*	%	*n*	%	*n*	%
Gender	Man	40	48.19	40	47.06	40	48.19
	Woman	43	51.81	45	52.94	43	51.81
Age Range	41–49	33	36.14	26	30.59	30	34.94
	50–57	26	34.94	40	47.06	27	33.73
	58–65	24	28.92	19	22.35	26	31.33
Educational Level	0–15 years	26	31.33	24	28.24	26	31.33
	≥16 years	57	68.67	61	71.76	57	68.67

We found no differences between the performance of the groups in any neuropsychological measure according to one-way ANOVA tests calculated. It is important to note that they did not differ in the RAVLT scores (see Supplementary Table 5 for details). Therefore, they are homogeneous groups in terms of their cognitive execution, especially in their performance on the RAVLT Immediate Total Recall, Delayed Recall, and Recognition.

Descriptives of the performance of the groups on the LTF measures (1-week Delayed Recall, 1-week Recognition, Intrusions, and 1-week Forgetting Rate) are presented in Table 12.

**TABLE 12:** Performance on LTF assessment.

	Group	Min	Max	Mean	SD
1-week Delayed Recall	I	1	14	6.48	3.01
	II	2	15	6.24	2.80
	III	1	15	7.64	3.28
1-week Delayed Recognition	I	6	15	12.55	1.97
	II	7	15	12.58	1.85
	III	6	15	12.77	1.91
Intrusions	I	0	5	0.66	1.11
	II	0	3	0.67	0.88
	III	0	5	0.69	0.96
1-week Forgetting Rate	I	0	87.5	46	20.22
	II	0	83.33	46.29	17.50
	III	−9.09	88.89	35.80	20.67

Regardless of application modality, the participants evoked fewer words from the list in the 1-week delayed recall than the recall trial 25 min after initial learning (Delayed Recall), resulting statistically significant in the calculated mixed ANOVA [Delay main effect: *F*(1121.91), *p* < 0.001, ηp^2^ = 0.82].

**FIGURE 1:**
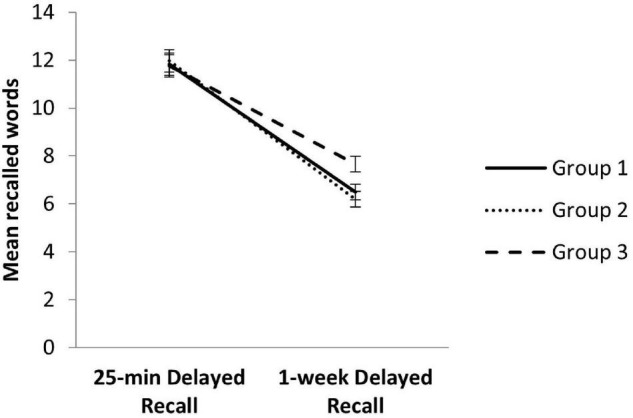
Performance on 25-min Delayed Recall and 1-week Delayed Recall between modality groups. Error bars represent SEM.

The interaction Delay × Group also resulted significant (*F* = 6.99, *p* = 0.001, ηp^2^ = 0.05), indicating differences in the RAVLT performance between the three groups depending on the deferred lapse after initial learning. As Figure 1 illustrates, the decrease in the number of evoked words between the 25-min and 1-week delayed recall measures depends on the application modality.

*Post hoc* analysis revealed no differences between the three groups in the 25-min Delayed Recall (*F* = 0.44, *p* = 0.64), as we previously demonstrated through the one-way ANOVA, but 1-week delayed measures showed significant differences (*F* = 5.09, *p* = 0.01). Specifically, the participants of Group III, who were explicitly warned about the 1-week recall, performed significantly better than the participants of Group I (*p* = 0.04) and Group II (*p* = 0.01) (see Figure 1).

Finally, multiple regression linear analyses were carried out for each modality group to determine the contribution of demographic variables as predictors of 1-week delayed measures. Concerning Group I, the regression model [*F*(3,79) = 6.97; *p* < 0.001] reflected that age (β = −0.42; *p* < 0.001) and gender (β = −0.20; *p* = 0.04) explained 21% of the variance of 1-week Delayed Recall. Men and older participants tended to recall fewer words after 1-week. For their Recognition score [*F*(3,79) = 3.14; *p* = 0.02; *R^2^* = *0.12*], only age appeared as a significant predictor (β = −0.27; *p* = 0.02). Also, 12% of the variance of Forgetting Rate [*F*(3,79) = 3.51; *p* = 0.20] was predicted by age (β = 0.31; *p* = 0.01), showing that younger participants tend to forget less.

Regarding Group II, the regression model for 1-week Delayed Recall [*F*(3,81) = 6.34; *p* < 0.001; *R^2^* = *0.19*] indicated that gender was the only predictor (β = −0.44; *p* < 0.001), and Recognition was explained [*F*(3,81) = 2.96; *p* = 0.04; *R^2^* = *0.10*] by years of education (β = 0.28; *p* = 0.01; *R^2^* = *0.10*). These findings reflect that women within this group tend to spontaneously recall more words, while those with lower educational levels obtain poorer performance in the recognition task. With respect to Forgetting Rate [*F*(3,81) = 3.90; *p* = 0.01; *R^2^* = *0.01*], gender was the only statistically significant predictor (β = 0.35; *p* < 0.001), confirming that men tend to evoke fewer words after 1-week.

Finally, for Group III, the 1-week Delayed Recall regression model [*F*(3,79) = 3.03; *p* = 0.03; *R^2^* = *0.10*] was the only statistically significant, with gender being the predictor of the scores (β = −0.22; *p* = 0.04). The findings reflected that men tended to perform worse.

## Discussion

Episodic memory (EM) assessment is a fundamental pillar in the study of brain aging that provides premature signs of neurodegenerative disorders due to its multidimensional complexity and high vulnerability to disease (Pause et al., 2013; Polcher et al., 2017). Even in cognitively normal older adults, an association between amyloid burden and episodic memory performance has been detected. Therefore, the earliest preclinical stages of dementia may have subtle but measurable effects on cognition that could help identify diseases prematurely (Hedden et al., 2012; Duke Han et al., 2017; Baker et al., 2018).

In this sense, the RAVLT is a potential sensitive tool to measure changes associated with abnormal aging (Estévez-González et al., 2003; Drolet et al., 2014; Marchand et al., 2017; Moradi et al., 2017). Thus, this test has been widely used in longitudinal studies to help describe the memory trajectory in cognitively normal and MCI subjects as an indicator of dementia risk. Sum Trials I to V, the RAVLT learning score (V-I), and 25-min delayed recall play an important role in the prediction of progression from MCI to AD dementia (Crane et al., 2012; Wang et al., 2016; Li et al., 2017; Li et al., 2018; Ding et al., 2019; Abraham et al., 2020). Also, error scores have been associated with different types and stages of cognitive impairment (Baños et al., 2005; Thomas et al., 2018; Weitzner et al., 2020). For this reason, considering that preclinical changes appear up to two decades before the diagnosis of AD dementia (Chipi et al., 2019), it is relevant and indispensable to have sensitive tests with normative data for the middle-aged population, which allow comparisons between risk population (i.e., APOE carriers) and their corresponding normative values. Thus, a better understanding of preclinical and prodromal stages of AD would be obtained so early therapeutic interventions could be applied to prevent disability (Ritchie et al., 2017).

### Rey Auditory Verbal Learning Test Validation

Research on RAVLT construct validity through factor structure has been scarce, and most of the studies lacked the inclusion of error scores. It has been recommended to replicate the structural analysis in different samples and include repeated words and intrusions, considering their value (Vakil and Blachstein, 1993; Baños et al., 2005; Weitzner et al., 2020). Considering that no test validation has been performed in the healthy Spanish middle-aged population and the addition of error scores, it was necessary to perform an EFA to find out how many factors can be extracted after including error scores. Then, to finally determine the factor structure of the test and its goodness of fit, we carried out CFA through the rival model strategy (Hair et al., 1999; Lloret-Segura et al., 2014).

Our CFA results showed that the model with the best goodness of fit is the four-factor model, which includes the first component associated with the Attention and Memory span (Trials I and II), the second one related to Memory and Learning (Trials III, IV, and V, Delayed Recall, and Recognition), and the third and fourth referring to error measures such as Perseverations and Intrusions, respectively. The resulting factor structure corresponds to the theoretical framework and previous findings, although some of those studies did not include error measures, unlike our proposal.

Vakil and Blachstein (1993) found a factor structure formed by the three main memory processes (Squire, 1982; Lezak et al., 2012): acquisition, storage, and retrieval. Acquisition (Trial I) is related to the attention and memory span, Retrieval includes the learning score and delayed recall trial, and Storage relates to the recognition score only. This solution resembles the one shown in the present study, although we found that the recognition score loaded within the Memory and Learning factor. Vakil and Blachstein (1993) emphasized that memory components, in normal subjects, would be strongly related to each other, explaining why the recognition and consolidation processes grouped.

In addition, Baños et al. (2005) obtained a three-factor solution that includes a significant factor indexing auditory attention, another relative to verbal learning, and the last one with inaccurate recall loaded (only intrusions), consistent with our findings. Finally, Weitzner et al. (2020) found a factorial solution with a good fit in a middle-aged sample similar to the one we described. However, they, in addition to the error measures, included other process scores. They defined the Attention/Learning factor that partially corresponds to our first component (the Attention and Memory span) even though we did not consider serial position scores. The Memory factor, related to short-term memory, long-term memory, and recognition, is comparable to our second component (Memory and Learning). Inaccurate Memory corresponds to our Repeated and Intrusion factors.

Our findings highlight the value of using multifactorial measures, including execution errors, to facilitate the characterization of healthy and clinical populations through an empirical framework for diagnosis, rehabilitation, or research purposes (Vakil and Blachstein, 1993; Baños et al., 2005; Weitzner et al., 2020). The quantification of error scores is an added worth, considering previous evidence of its predictive value of abnormal aging (Bezdicek et al., 2014; Ferreira Correia and Campagna Osorio, 2014; Libon et al., 2015; Thomas et al., 2018; Weitzner et al., 2020). Thus, we firmly recommend including these measures and other process measures in clinical and longitudinal studies to confirm their discriminative and predictive capacity in early aging.

Regarding convergent validity, Fichman et al. (2010) found a positive linear relation between the RAVLT and a memory task of the Brief Cognitive Screening Battery (BCSB), even though the input of this test is visual. In the present study, we found a positive correlation with the S-FNAME Exam, an associative memory test of verbal-visual stimuli that require immediate and delayed recall, with more demanding encoding, storage, and retrieval strategies than BCSB used in previous research. As a limitation, The FNAME and the RAVLT are instruments that correspond to different paradigms; both cover verbal stimuli, but the FNAME is a visual EM test. Therefore, we recommend assessing the convergent validity using verbal auditory EM tests in Spanish for future research.

According to divergent validity, weak positive correlations between the RAVLT and non-amnesic tests have been demonstrated. These results were also significantly lower than the linear relation with the S-FNAME Exam. Our findings are similar to previous results (de Paula et al., 2012), highlighting that the RAVLT assesses a different construct than those measured by widely used non-amnesic tests.

### Rey Auditory Verbal Learning Test Norms Development

In line with previous findings, women outperformed males on RAVLT scores, especially on immediate total recall and delayed recall (Gale et al., 2007; Messinis et al., 2007; Speer et al., 2014). Delayed RAVLT recall in elderly samples suggests that men remain stable across age ranges, while women show a subtle decline. Differences in verbal memory by sex may be related to distinct encoding and consolidation strategies (Gale et al., 2007; Zhang et al., 2017).

It has been widely demonstrated that age has an inverse relation with EM (Schoenberg et al., 2006; Fichman et al., 2010; Vakil et al., 2010; Marqués et al., 2013; Ferreira Correia and Campagna Osorio, 2014; Speer et al., 2014; Lavoie et al., 2018). However, not all components of EM change homogeneously with age. Older adults reflect recall impairments but typically showed minimal age decline in recognition tasks (Healey and Kahana, 2016). This divergence in trajectories is consistent with our RAVLT findings: Age does not significantly influence recognition, but affects immediate and delayed recall. Regarding errors, unlike our results, Baños et al. (2005) reported lower inaccurate recall scores for younger individuals.

Finally, the influence of education on RAVLT scores is widely described. The higher educational level has been associated with better performance as we found in the present study, although some previous studies did not find the significative contribution of this variable (Messinis et al., 2007; Fichman et al., 2010; Ferreira Correia and Campagna Osorio, 2014; Lavoie et al., 2018). Understanding the effect of demographic variables on RAVLT and verbal episodic memory is essential for designing prevention, stimulation, and rehabilitation protocols in aging.

Neuropsychological assessment using standardized neurocognitive measures is a priority within clinical and research practice. Thus, developing sensitive measures to identify the early stages of neurocognitive disorders is a current need to plan a comprehensive treatment (Moradi et al., 2017). There is still a need to develop and publish normative data for Spanish-speaking subjects, considering that cultural, linguistic, or sociodemographic variables could affect a cognitive profile and vary between different populations (Peña-Casanova et al., 2012; Del Pino et al., 2015).

The exclusion criteria of the present study were chosen to ensure a sample of cognitively healthy subjects. However, the limitation of the study is that it lacks subjective complaints and biological measures that could identify participants within the preclinical phase of AD. Regarding the age range, it is essential to highlight that the objective of the present study was to validate the Spanish version of the RAVLT and generate normative data for the middle-aged population, considering the lack of psychometric studies for this population in Spain. Marqués et al. (2013) published normative data for a Spanish version of the RAVLT in older people, but it is crucial to have a memory test that allows early measurement of changes associated with aging. Also, we strongly recommend replicating the factorial structure using an older aged sample and extending normative data and evidence of validity in other age ranges.

Our sample size was large enough to be representative and met the criteria for validation studies and factor analysis requirements (Hair et al., 1999; Speer et al., 2014). Nevertheless, the overrepresentation of highly educated subjects in our cognitively normal sample should be considered a limitation. This unbalanced distribution by the educational level is frequent in other Spanish normative studies (Del Pino et al., 2015) and has been reported in previous RAVLT validation projects (Lavoie et al., 2018; Weitzner et al., 2020). Then et al. (2016) suggested, based on the exploration of the relationship between dementia risk and education, that strongest prediction of low risk of dementia is obtained by the stratification in low (non-tertiary education) and high (tertiary education) educational levels.

Different RAVLT versions with variations administration procedures have been developed (Schmidt, 1996; Estévez-González et al., 2003; Lezak et al., 2012; Bezdicek et al., 2014; Cavaco et al., 2015; Puerta Lopera et al., 2018). It is crucial to notice that the Spanish RAVLT version used lacks Interference List B, which provides information on the effect of interference on memory consolidation. This version is frequently used in Spain (Perea Bartolomé et al., 2000; Estévez-González et al., 2003; García-Rudolph et al., 2020; Albu et al., 2021); however, this may be considered a limitation and should be taken into account when using these normative data. As Estévez-González et al. (2003) and Messinis et al. (2007) mentioned, the RAVLT application methods have not been uniform in the literature, sometimes restricting the use of normative data.

### Long-Term Forgetting Assessment

Beyond these limitations discussed above, a relatively novel and potentially vital aspect of this study is the introduction of LTF assessment, a sensitive marker of memory decline in abnormal aging (Reiman, 2018; Weston et al., 2018; Zimmermann and Butler, 2018; Butler et al., 2019; Wearn et al., 2020). Clinical and theoretical interest in LTF has emerged recently, and it is recommended to use more demanding cognitive instruments, including LTF measures, to detect the earliest cognitive manifestations in presymptomatic AD (Butler et al., 2019; Tort-Merino et al., 2021a).

Considering methodological issues exposed by Elliott et al. (2014) associated with the procedures and rehearsal effects in longitudinal follow-ups, the present study compared the performance on RALTV, adding 1-week delayed retrieval after the initial learning, under three different procedures. Within the framework of long-term forgetting assessment, this is the first study, to our knowledge, that shows evidence about the difference between warning the participants or not about the delayed recall with a more extended period since the initial learning.

Results revealed that, while there were no differences in the immediate and 25-min delayed recall between the three groups, the participants informed about the 1-week recall test showed less LTF, extending prior findings that revealed the influence of test expectations have on the establishment of new memories in long-term storage (Shimizu, 1996; Szpunar et al., 2007). The “expectancy manipulation” was done after initial learning, excluding the possible effect on encoding. These results suggest that consolidation is associated with top-down modulation by knowing the value and utility of the previously learned information (Wamsley et al., 2016). Retroactive interference related to mental effort and learning of new material may explain the decay of memory due to the participation of the hippocampus in the consolidation phase (Hoefeijzers et al., 2013; Brawn et al., 2018). Retroactive interference was not controlled in the present study and should be considered in future LTF research. Also, it is essential to consider that, although the participants were asked not to practice, there is a possibility that they have done so. Information retrieval permits memory integration with stored neocortical knowledge, making memory less hippocampus dependent and more reachable for recall in the future (Antony et al., 2017). That is why it is challenging to design a genuinely unexpected test more than one time and to control the effect of rehearsal through a longitudinal assessment (Lee et al., 2017; Wyble and Chen, 2017).

On the other hand, the performance of Group I subjects, who were unaware of the LTF assessment, could be a reliable measure of incidental long-term recall, considering that all of them claimed not to have practiced, as previously mentioned. It would be interesting to determine the performance of these subjects on repeated longitudinal measures to expand knowledge regarding the LTF construct, since given the delayed recall experience, possible test expectations would be generated.

In any of the three modalities, we present a measurement approach that encompasses both delayed free recall and a recognition task. These memory paradigms are fundamental to understanding LTF because the cognitive profiles of clinical samples may reflect differences in performance on these two tasks, as noted by Elliott et al. (2014).

It is relevant to point out that previous LTF research (Weston et al., 2018; Zimmermann and Butler, 2018) has fixed a minimum acquired criterion (accuracy of 80% over a maximum of 10 initial trials), considering forgetting rates are inherently related to learning performance. Considering that this administration procedure would have altered the RAVLT version used in this validation study, we did not follow this criterion, as described in the Section “Materials and Methods.” However, we excluded subjects with objective cognitive deficits during the neuropsychological assessment, including the Immediate Total Recall. Furthermore, we demonstrated no differences in the Immediate Total Recall score between the three groups, and their performance reflected an accuracy of 70%.

This study highlights the importance of knowing the effect of different methodologies in memory assessment and the urgency of having agreed on methodological procedures to interpret LTF data and compare findings without bias. More studies on LTF should be done in the preclinical stages of abnormal aging pathologies to confirm their predictive value and describe possible relations with other biomarkers (Wearn et al., 2020).

Hence, it is crucial to design or adapt valid LTF instruments (Wearn et al., 2020), even for the middle-aged population, establishing best practices that minimize potential rehearsal and learning effects associated with longitudinal assessments. Also, it is urgent to provide normative data with a broader sample, including long-term recall measures after 4 weeks or 3 months, considering previous findings that relate these measures to AD biomarkers (Tort-Merino et al., 2017, 2021a,2021b; Wearn et al., 2020). The availability of LTF instruments would be beneficial in improving the sensitivity of conventional EM tests in both aging research and clinical practices.

## Conclusion

In conclusion, the validation and normalization of the RAVLT in a Spanish sample and the proposal of an LTF measure using this widely known instrument are extremely valuable, considering that the RAVLT is a helpful tool, along with other biomarkers, to develop predictive models of healthy and pathological aging. It is recommended to conduct studies with subjects within the preclinical phase of AD, and patients with MCI and AD diagnosis, to detect its sensitivity, specificity, and precise cut-off points that reflect a cognitive decline (Reiman, 2018). Also, we suggest including APOE status or other AD markers in plasma or CSF to characterize the sample and determine the relationship between RAVLT and these biological measures.

## Data Availability Statement

The raw data supporting the conclusions of this article will be made available by the authors, without undue reservation.

## Ethics Statement

The studies involving human participants were reviewed and approved by Comité d’Ètica i Investigació Clínica de la Unió Catalana d’Hospitals. The patients/participants provided their written informed consent to participate in this study.

## Author Contributions

GC, DB-F, and VA-S participated in drafting the manuscript and made substantial contributions to the analysis and interpretation of data. CP-G, GC, and VA-S made substantial contribution to the acquisition of data. All authors made substantial contributions to conception, design, and interpretation of data, contributed to revising it critically for important intellectual content, and approved the submitted version.

## Conflict of Interest

The authors declare that the research was conducted in the absence of any commercial or financial relationships that could be construed as a potential conflict of interest.

## Publisher’s Note

All claims expressed in this article are solely those of the authors and do not necessarily represent those of their affiliated organizations, or those of the publisher, the editors and the reviewers. Any product that may be evaluated in this article, or claim that may be made by its manufacturer, is not guaranteed or endorsed by the publisher.
