# Supplementary material for: Validation and Normative Data of the Spanish Version of the Rey Auditory Verbal Learning Test and Associated Long-Term Forgetting Measures in Middle-Aged Adults
Source: Front Aging Neurosci. 2022 Feb 9;14:809019. doi: 10.3389/fnagi.2022.809019 (PMC8865334; doi:10.3389/fnagi.2022.809019)
Supplement: Supplementary file 1 [file Data_Sheet_1.pdf]

Supplementary Material

Table S1. Goodness of fit indices of CFA

	Absolute fit índices				Incremental fit indices			Parsimonious fit indices	
**Factorial structure**	**χ^2**	**χ^2/gl**	**GFI**	**RMSEA**	**AGFI**	**NFI**	**TLI**	**PNFI**	**PGFI**
**2-factor model**	160.46	6.17	0.92	0.09	0.87	0.72	0.66	0.52	0.53
**3-factor model**	180.81	5.48	0.92	0.09	0.87	0.71	0.66	0.53	0.55
**4-factor model**	109.50	3.65	0.95	0.07	0.92	0.83	0.80	0.55	0.52

Notes: χ^2= Chi-square; χ^2/df= Normed Chi-square; GFI= Goodness of Fit Index; RMSEA= Root Mean Square Error of Approximation; AGFI= Adjusted Goodness Fit Index; NFI= Normed Fit Index; TLI= Tucker-Lewis Index*;* PNFI= parsimonious normed fit index; PGFI= Parsimony Goodness-of-Fit Index

Table S2**.** Correlation coefficients between RAVLT scores and sociodemographic variables

	**Age**	**Years of education**
**I**	-0.19**	0.10*
**II**	-0.21**	0.11**
**III**	-0.24**	0.12**
**IV**	-0.23**	0.15**
**V**	-0.20**	0.15**
**Immediate Total Recall**	-0.26**	0.15**
**Recognition**	-0.12**	0.15**
**Delayed Recall**	-0.21**	0.16**
**Repeated words**	0.05	0.01
**Intrusions words**	-0.01	-0.10*
**Repetead intrusions**	0.02	-0.10*
**LOT**	0.06	0.05
**Forgetting Rate**	.011**	-0.08*

* $p < .05$ ** $p < .01$

Notes: I = Trial I of Rey Auditory Verbal Learning Test (RAVLT); II = Trial II of RAVLT; III = Trial III of RAVLT; IV = Trial IV of RAVLT; V = Trial V of RAVLT; LOT= Learning Over Trial

Table S3. Performance on Immediate Total Recall and Delayed Recall according to gender, age and education

Sociodemographic variables			RAVLT scores	
Gender	*Age Ranges*	*Years of Education*	Immediate Total Recall Mean (SD)	Delayed Recall Mean (SD)
Women	41-49	< 16 years	51.86 (6.93)	11.19 (2.46)
		≥16 years	57.10 (7.19)	12.62 (2.41)
	50-57	< 16 years	52.52 (7.58)	11.81 (2.11)
		≥16 years	54.09 (7.97)	12.03 (2.16)
	58-65	< 16 years	51.41 (8.45)	11.27 (2.65)
		≥16 years	52.83 (7.59)	11.33 (2.31)
Men	41-49	< 16 years	52.44 (6.82)	11.07 (2.34)
		≥16 years	53.88 (7.65)	11.90 (2.34)
	50-57	< 16 years	48.62 (7.63)	10.15 (2.75)
		≥16 years	51.22 (8.45)	11.25 (2.68)
	58-65	< 16 years	46.18 (7.43)	9.90 (2.23)
		≥16 years	48.10 (7.61)	10.25 (2.79)

Table S4. Regression-based equations to calculate RAVLT z-scores

RAVLT score	Equations
**Immediate Total Recall**	z = [Observed raw score - 64.14 - 0.29(age) - 3.56(sex) + 0.29(years of education)]/7.67
**Delayed Recall**	z = [Observed raw score - 13.89 - 0.29(age) - 0.97(sex) + 0.100(years of education)]/2.45

Table S5. Description of LTG Groups performance in neuropsychological assessment protocol

Neuropsychological measure	Group	Mean	SD	F	Sig.
	I	16.80	9.90		
S-FNAME FN-N	II	18.64	10.78	1.24	0.29
	III	16.01	10.94		
	I	26.36	9.25		
S-FNAME FN-O	II	26.09	10.11	0.02	0.98
	III	26.37	10.37		
	I	6.05	1.13		
Direct digit span	II	6.05	1.29	0.04	0.96
	III	6.10	1.30		
	I	4.78	1.05		
Inverse digit span	II	4.86	.99	2.14	0.12
	III	5.10	1.02		
	I	26.78	9.56		
TMT A	II	25.65	6.75	1.40	0.25
	III	27.77	8.19		
	I	77.14	22.74		
TMT B	II	76.94	21.65	0.46	0.63
	III	79.96	24.10		
	I	18.19	4.57		
Phonemic Fluency	II	18.18	4.05	0.01	0.99
	III	18.28	4.49		
	I	25.06	5.36		
Semantic Fluency	II	24.26	5.13	1.61	0.20
	III	23.63	5.01		
	I	20.28	3.39		
Reasoning Matrix	II	20.06	3.03	0.10	0.91
	III	20.12	3.57		
Digit-Symbol	I	78.42	13.17	1.43	0.24

	**II**	81.12	11.91		
	**III**	78.11	13.02		
**Immediate Total Recall RAVLT**	**I**	52.64	8.37	0.44	0.64
	**II**	52.41	8.73		
	**III**	53.58	8.43		
**Delayed Recall RAVLT**	**I**	11.80	2.45	0.56	0.58
	**II**	11.41	2.49		
	**III**	11.73	2.67		
**RAVLT Recognition**	**I**	14.39	1.32	0.34	0.71
	**II**	14.49	1.02		
	**III**	14.52	.92		
**Corsi block tapping test**	**I**	6.65	.97	0.731	0.48
	**II**	6.49	.96		
	**III**	6.49	.97		
**Block desing**	**I**	46.71	10.24	0.11	0.89
	**II**	46.18	9.82		
	**III**	45.98	10.86		
**Letter-Number sequency**	**I**	5.71	.99	2.92	0.06
	**II**	5.46	1.09		
	**III**	5.84	1.07		
**Cancellation**	**I**	41.83	9.15	0.01	0.99
	**II**	41.92	8.07		
	**III**	41.95	9.46		

Notes: S-FNAME FN-N= subtotal face-name association of Spanish FNAME Exam; S-FNAME FN-O = subtotal face-occupation association of Spanish FNAME Exam; TMT-A = Trail Making Test part A; TMT-B = Trail Making Test part B
